# Artificial intelligence assistance significantly improves Gleason grading of prostate biopsies by pathologists

**DOI:** 10.1038/s41379-020-0640-y

**Published:** 2020-08-05

**Authors:** Wouter Bulten, Maschenka Balkenhol, Jean-Joël Awoumou Belinga, Américo Brilhante, Aslı Çakır, Lars Egevad, Martin Eklund, Xavier Farré, Katerina Geronatsiou, Vincent Molinié, Guilherme Pereira, Paromita Roy, Günter Saile, Paulo Salles, Ewout Schaafsma, Joëlle Tschui, Anne-Marie Vos, ISUP Pathology Imagebase Expert Panel, Brett Delahunt, Hemamali Samaratunga, David J. Grignon, Andrew J. Evans, Daniel M. Berney, Chin-Chen Pan, Glen Kristiansen, James G. Kench, Jon Oxley, Katia R. M. Leite, Jesse K. McKenney, Peter A. Humphrey, Samson W. Fine, Toyonori Tsuzuki, Murali Varma, Ming Zhou, Eva Comperat, David G. Bostwick, Kenneth A. Iczkowski, Cristina Magi-Galluzzi, John R. Srigley, Hiroyuki Takahashi, Theo van der Kwast, Hester van Boven, Robert Vink, Jeroen van der Laak, Christina Hulsbergen-van der Kaa, Geert Litjens

**Affiliations:** 1 Department of Pathology, Radboud Institute for Health Sciences, Radboud University Medical Center, Nijmegen, The Netherlands; 2 Department of Morphological Sciences and Anatomic Pathology Faculty of Medicine and Biomedical Sciences, University of Yaounde 1, Yaounde, Cameroon; 3 Salomão Zoppi Diagnostics/DASA, São Paulo, Brazil; 4 Pathology Department, School of Medicine, Istanbul Medipol University, Istanbul, Turkey; 5 Department of Oncology and Pathology, Karolinska Institutet, Stockholm, Sweden; 6 Department of Medical Epidemiology and Biostatistics, Karolinska Institutet, Stockholm, Sweden; 7 Department of Health, Public Health Agency of Catalonia, Lleida, Catalonia Spain; 8 Centre de Pathologie, Hopital Diaconat Mulhouse, Mulhouse, France; 9 Pathology department, Aix en Provence Hospital, Aix-en-Provence, France; 10 Histo Patologia Cirúrgica e Citologia, João Pessoa-PB, Brazil; 11 Department of Pathology, Tata Medical Center, Kolkata, India; 12 Iabor team w ag, Abteilung für Histopathologie und Zytologie, Goldach SG, Switzerland; 13 Instituto Mário Penna, Belo Horizonte, Brazil; 14 Medics Pathologie, Bern, Switzerland; 15 Department of Pathology, Antoni van Leeuwenhoek Hospital, The Netherlands Cancer Institute, Amsterdam, The Netherlands; 16 Laboratory of Pathology East Netherlands, Hengelo, The Netherlands; 17 Center for Medical Image Science and Visualization, Linköping University, Linköping, Sweden; 18 Department of Pathology and Molecular Medicine, Wellington School of Medicine and Health Sciences, University of Otago, Wellington, New Zealand; 19 Aquesta Uropathology and University of Queensland, Brisbane, QLD Australia; 20 Department of Pathology and Molecular Medicine, Indiana University School of Medicine, Indianapolis, IN USA; 21 Laboratory Medicine Program, University Health Network, Toronto General Hospital, Toronto, ON Canada; 22 Barts Cancer Institute, Queen Mary University of London, London, UK; 23 Department of Pathology, Taipei Veterans General Hospital, Taipei, Taiwan; 24 Institute of Pathology, University Hospital Bonn, Bonn, Germany; 25 Department of Tissue Pathology and Diagnostic Oncology, Royal Prince Alfred Hospital and Central Clinical School, University of Sydney, Sydney, NSW Australia; 26 Department of Cellular Pathology, Southmead Hospital, Bristol, UK; 27 Department of Urology, Laboratory of Medical Research, University of São Paulo Medical School, São Paulo, Brazil; 28 Pathology and Laboratory Medicine Institute, Cleveland Clinic, Cleveland, OH USA; 29 Department of Pathology, Yale University School of Medicine, New Haven, CT USA; 30 Department of Pathology, Memorial Sloan-Kettering Cancer Center, New York, NY USA; 31 Department of Surgical Pathology, School of Medicine, Aichi Medical University, Nagoya, Japan; 32 Department of Cellular Pathology, University Hospital of Wales, Cardiff, UK; 33 Department of Pathology and Laboratory Medicine, Tufts Medical Center, Boston, MA USA; 34 Hôpital Tenon, HUEP, AP‐HP, UPMC Paris VI, Sorbonne Universities, Paris, France; 35 Bostwick Laboratories, Orlando, FL USA; 36 Department of Pathology, Medical College of Wisconsin, Milwaukee, WI USA; 37 Department of Anatomic Pathology, Cleveland Clinic Lerner College of Medicine, Cleveland Clinic, Cleveland, OH USA; 38 Department of Laboratory Medicine and Pathobiology, University of Toronto, Toronto, ON Canada; 39 Department of Pathology, Jikei University School of Medicine, Tokyo, Japan

**Keywords:** Prostate cancer, Software

## Abstract

The Gleason score is the most important prognostic marker for prostate cancer patients, but it suffers from significant observer variability. Artificial intelligence (AI) systems based on deep learning can achieve pathologist-level performance at Gleason grading. However, the performance of such systems can degrade in the presence of artifacts, foreign tissue, or other anomalies. Pathologists integrating their expertise with feedback from an AI system could result in a synergy that outperforms both the individual pathologist and the system. Despite the hype around AI assistance, existing literature on this topic within the pathology domain is limited. We investigated the value of AI assistance for grading prostate biopsies. A panel of 14 observers graded 160 biopsies with and without AI assistance. Using AI, the agreement of the panel with an expert reference standard increased significantly (quadratically weighted Cohen’s kappa, 0.799 vs. 0.872; *p* = 0.019). On an external validation set of 87 cases, the panel showed a significant increase in agreement with a panel of international experts in prostate pathology (quadratically weighted Cohen’s kappa, 0.733 vs. 0.786; *p* = 0.003). In both experiments, on a group-level, AI-assisted pathologists outperformed the unassisted pathologists and the standalone AI system. Our results show the potential of AI systems for Gleason grading, but more importantly, show the benefits of pathologist-AI synergy.

## Introduction

The biopsy Gleason score is the most important tissue-based prognostic marker for prostate cancer patients [1]. However, it has been shown that Gleason grading suffers from significant inter- and intraobserver variability [2, 3]. Specialized uropathologists show higher concordance rates [4], but such expertise is not always available. Artificial intelligence (AI) systems based on deep learning have achieved pathologist-level performance in Gleason grading [5–8], but it is not yet investigated whether pathologists improve in Gleason grading if they are assisted by such systems.

Pathologists assess the Gleason grade of a prostate biopsy through microscopic assessment of tissue stained with hematoxylin and eosin (H&E). Based on the morphological pattern of the tumor, a grade between one and five is assigned, with one being the lowest and five the highest. For biopsies, the Gleason score is the sum of the two most common patterns, e.g., 3 + 5 = 8. If a higher tertiary pattern is present, this is used instead of the secondary pattern. Patterns 1 and 2 are not reported anymore for biopsies [9].

Recently, (ISUP) grade groups were introduced which aimed to improve the reporting of Gleason grading by assigning the Gleason score to one of five prognostic groups [10]. These groups are directly based on the Gleason score; 3 + 3 and lower go to group 1, 3 + 4 to group 2, 4 + 3 to group 3, 3 + 5, 5 + 3 and 4 + 4 to group 4, and higher scores to group 5. While the introduction of grade groups showed clinical value and increased interpretability of the tumor grade for patients, it has not improved the inter- and intraobserver variability [5, 11].

Deep learning has shown promise in many medical fields [12], and the introduction of digital pathology allows for AI-based diagnostics in pathology [13]. For prostate cancer, methods based on deep learning have been developed for tumor detection [7, 8, 14–16], grading of prostatectomies [5], tissue microarrays [6], and biopsies [7, 8, 17]. In multiple studies, such deep learning systems showed pathologist-level performance, within the limits of the study setup [5, 7, 8].

Although deep learning systems have shown to achieve high performances on grading tasks, evidence of the merit of such systems when embedded in the pathologist’s workflow is limited. Deep learning systems can be viewed as a new tool for pathologists to use in their diagnostic process and should also be evaluated as such. In addition, regardless of the merits, most developed systems are also constrained by significant limitations that affect the performance and can lower the diagnostic power. Within histopathology, the presence of non-prostate tissue, atypical tissue patterns, ink on a slide, fixation, scanning and cutting artifacts, or the presence of rare cancer subtypes can dramatically affect a system’s assessment of tissue. Many of these errors, especially those caused by artifacts, are easily spotted by a human observer.

Studies combining experts’ opinions with feedback from automated systems have mainly been performed outside of the field of pathology; for example on the task of breast cancer detection in mammography [18]. For pathology, on the task of cancer metastasis detection in lymph nodes, the sensitivity of detection of micrometastases increased, and overall case reading time went down as a result of AI support [19]. On the task of mitosis counting, AI-generated hotspots improved reproducibility between readers [20]. For prostate cancer, AI assistance has shown potential in increasing sensitivity for detecting cancer in biopsies [16]. However, most of these studies focus either on computer-aided detection or diagnosis. For prognostic measures, such as Gleason grading of prostate biopsies, there is, to the best of our knowledge, no such study as of yet.

In a previous study, we developed a fully automated deep learning system for grading prostate cancer [7]. The deep learning system was trained on a large dataset of prostate biopsies and achieved pathologist-level performance, both in determining the grade group and in stratifying patients in relevant risk categories. As part of the initial validation of the system, its performance was compared with a panel of pathologists in an observer experiment. The deep learning system outperformed 10 out of 15 observers on determining the grade group.

In this study, we investigate the value of AI-assisted reading by pathologists for Gleason grading of prostate biopsies by comparing the diagnostic performance of pathologists with and without the assistance of a deep learning system.

## Materials and methods

### Collection of the dataset and setting the reference standard

In a previous study [7], we developed a deep learning system to grade prostate biopsies using the Gleason grading system. To train this system, we collected a dataset of 5759 H&E-stained biopsies from 1243 patients. All biopsy procedures were performed as part of routine diagnostics at the Radboud University Medical Center between 2012 and 2017. For the study, the H&E-stained glass slides of the biopsies were digitized at 20× magnification (pixel resolution 0.24 µm) using a 3DHistech Pannoramic Flash II 250 scanner and subsequently anonymized. The need for informed consent was waived by the local ethics review board (IRB number 2016–2275).

Of the dataset, 550 biopsies were excluded from model development and used as an independent test set to evaluate the deep learning system. Patients that were included in this test set were independent of the patients in the training set. Given the inter-observer variability of Gleason grading, validation of the system required a consensus reference standard. In the first round, three expert pathologists (CH-vdK, RV, HvB) with a subspecialty in uropathology individually graded the cases in the test set using the ISUP 2014 guidelines [21]. For some cases the majority vote was taken: cases with an agreement on grade group but a difference in Gleason pattern order, e.g., 5 + 4 versus 4 + 5; cases with an equal grade group but a disagreement on Gleason score; and cases for which two pathologists agreed while the third had a maximum deviation of one grade group. Cases with a disagreement on malignancy were always flagged. In the second round, cases that had no agreement were presented to the pathologist who deviated the most from the other two. Additional to the pathologist’s score, the scores of the two other pathologists were shown anonymously. Finally, biopsies without agreement after two rounds were discussed in a consensus meeting.

### Observer panel and case selection

Part of the first study was a comparison of the deep learning system to a panel of pathologists. Of the full test set, 100 cases were selected and presented to a panel of 13 external pathologists and two pathologists in training. Of these 100 cases, 20 benign cases were selected by one of the expert pathologists (CH-vdK). The benign cases were chosen to cover the full spectrum of possible pitfalls for cancer, including partial atrophy, reactive atypia, granulomatous inflammation with epithelioid cells, atypical adenomatous hyperplasia as well as HGPIN (Table 1). The other 80 cases were sampled based on the grade group assignment by the same pathologist, selecting an equal number of cases per grade group. Potential pitfalls in the set of malignant cases are shown in Table 2. The panel was asked to grade all biopsies through an online viewer PMA.view (Pathomation, Berchem, Belgium) following the ISUP 2014 guidelines. No time limit was set for the grading process.

**Table 1:** Description and presence of inflammation for the benign cases of the internal test set.

Case ID	Description	Inflammation	Case ID	Description	Inflammation
20	AAH	Mild	109	RA + BCH + A	Mild
21	PA	Mild	113	none	None
23	RA + A	Mild	171	PA + A	None
30	RA + BCH + A	Mild	192	RA + A	Mild
33	HGPIN + RA + A	Mild	227	PA + HGPIN	Minimal
36	RA	Minimal	249	RA	Moderate
38	RA + A	Mild	280	A + PA	Minimal
66	PA	Minimal	284	RA + PA	Mild
67	AAH	None	287	HGPIN	None
68	A	None	326	PA + AAH	None
82	None	Minimal	333	A	None
88	RA	Moderate granulomatous	348	HGPIN + RA + PA	Minimal
90	RA	Severe granulomatous	398	RA + PA + BCH	Mild
94	PA	Mild	430	RA	Moderate
108	PA	Mild	482	PA	None

*A* (full) atrophy, *AAH* atypical adenomatous hyperplasia, *PA* partial atrophy, *RA* reactive atypia, *BCH* basal cell hyperplasia, *HGPIN* high grade PIN.

**Table 2:** Potential pitfalls in the set of malignant cases of the internal test set.

Case ID	Comment	Case ID	Comment
8	IDC dd HGPIN and G4 cribriform	277	IDC dd G4 cribriform
14	G5 dd -itis 3+	317	IDC dd HGPIN and G4 cribriform
112	G5 dd -itis 3+	370	IDC dd HGPIN and G4 cribriform
114	G5 dd -itis 3+	391	Partly foamy gland, no dd problem
132	Minimal cancer	395	Partly foamy gland, no dd problem
141	HGPIN dd G3	396	Partly foamy gland, no dd problem
186	Partly foamy gland, no dd problem	424	Foamy gland and IDC dd invasive
191	Partly foamy gland, no dd problem	439	IDC dd invasive G4
194	IDC dd HGPIN and G4 cribriform	448	Hyperplastic variant
212	Partly atrophic subtype	468	dd HGPIN vs. invasive
216	Foamy subtype	475	IDC dd HGPIN and G4 cribriform
229	IDC dd HGPIN and G4 cribriform	476	IDC dd G4 cribriform
234	Minimal cancer	526	IDC dd invasive G4
241	Minimal cancer and G5 dd -itis	529	HGPIN dd invasive G3
242	Partly hyperplastic type		

*IDC* intraductal carcinoma, *HGPIN* high grade PIN, *G3*, *G4*, *G5* growth pattern 3, 4, 5, *dd* differential diagnosis, -*itis* inflammation.

All pathologists that participated in the first study were invited to participate in the present study. One additional pathologist in training, who showed interest in the first study but was not able to grade all biopsies before submission of the previous paper, was also asked to join the current study. Panel members were not involved in the development of the AI system, nor had used the system before this study.

We included all 100 biopsies from the first study, as the panel already graded these cases. In addition, we extended the dataset with 60 new cases from the original test set, all of which were unseen by the panel members. These new unseen cases were used as control cases to measure the potential effect of a second-read on the original cases. One of the expert pathologists who set the reference standard (CH-vdK), selected ten benign cases manually, again controlling for a broad range of tissue patterns. The remaining fifty cases were sampled based on the consensus grade group, selecting an equal number of cases per group. All 160 biopsies were shuffled and assigned new identifiers. This dataset is further referenced as the internal dataset.

### Feedback of the AI system

We processed each biopsy in the dataset using the deep learning system [7], resulting in a prediction of the volume percentages of each Gleason pattern (if present), the Gleason score, and the grade group per biopsy. Besides a numerical prediction, the system also generated an overlay that outlined malignant glands: Gleason pattern 3 in yellow, Gleason pattern 4 in orange, and Gleason pattern 5 in red. For the current experiment, we chose not to highlight detected benign tissue. The overlays were postprocessed by a connected components algorithm to remove small artifacts and to ensure that each detected malignant gland was assigned to a single Gleason pattern. Postprocessing was done fully automatically without manual review. All selected biopsies were used, regardless of the correctness of the system’s prediction on these biopsies.

### Second-read with AI assistance

**Fig. 1:**
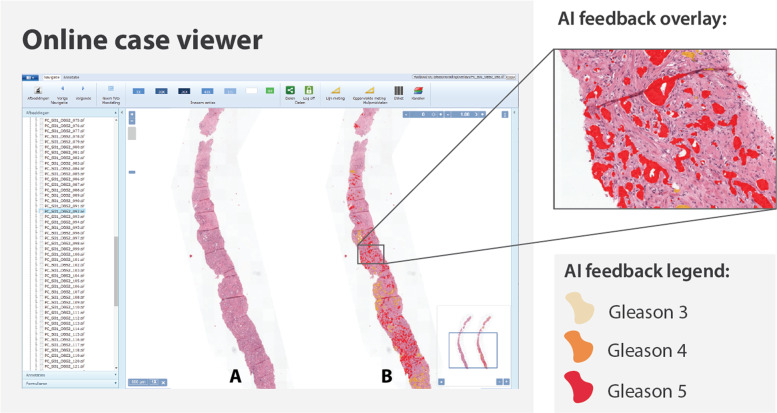
Overview of the viewer used in the observer experiment. Both the original biopsy (**a**) and the biopsy with the AI overlay (**b**) are presented to the pathologist. Each individual tumor gland is marked by the deep learning system in the overlay. The case-level grade group was supplied to the panel as part of their (separate) grading form.

The 160 biopsies were made available to the panel of pathologists through the same online viewer as during the first read. The time between the first and second read was at least 3 months. Each biopsy could be viewed at a maximum pixel spacing of 0.24 μm (roughly equivalent to 40× objective magnification). Next to the original biopsy, we showed an exact copy of the biopsy where AI-predicted Gleason patterns were highlighted using different colors (Fig. 1). This overlay could be used to assess the tissue that the algorithm had flagged as malignant. To complement the overlay, we also supplied the numerical output of the deep learning system to the panel, including the predicted volume percentages, the presence of tumor (yes/no), the Gleason score, and the grade group.

We asked each panel member to report: whether a biopsy contained tumor, presence of Gleason patterns, volume percentages of present patterns, and the grade group. After grading a case, the panel members had to indicate whether they thought the system’s prediction influenced their assessment.

No time restriction was given per case, but we asked each pathologist to complete all 160 cases within 8 weeks. Each panel member was instructed to review the cases individually without consulting colleagues. Panel members had no access to the cases from the previous experiment nor to the grades that they assigned. Between the first and second read, no performance indication or feedback was given to panel members with respect to the reference standard. When all cases were graded, each panel member was asked to fill in a questionnaire regarding the process and feedback from the deep learning system.

### External dataset

After the experiment on the internal dataset, we performed an additional experiment to test our hypothesis on external data. The time between the main experiment, and this external validation was 6 months. For this external validation, we made use of the Imagebase dataset [22]. The Imagebase set consists of 90 cases of prostate needle biopsies with cancer independently graded by 24 international experts in prostate pathology. Grading by the experts was done between May and September 2015, based on microphotographs taken from representative locations.

Each glass slide in the dataset consisted of two sections of the same biopsy, of which one was marked by pen. The biopsies were scanned on two different scanners as part of a previous independent study on automated Gleason grading by Ström et al. [8]. We extracted the marked biopsies and removed the pen markings. The deep learning system was applied as-is to the dataset without any normalization of the data. In the absence of a training set, the decision thresholds of the system were not optimized on the dataset. Instead, we determined that any detected tumor would classify the biopsy as malignant and used a 5% volume threshold for the inclusion of secondary patterns, comparable with clinical practice.

The setup of the experiment on the external dataset was equal to the experiment on the internal dataset. All pathologists who took part in the first experiment were invited to join the second experiment. Pathologists were given 1 week to grade the Imagebase cases. Instead of using microphotographs, pathologists were given access to the full biopsy through the digital viewer. After the unassisted read and a 2-week washout-period, the pathologists had to reexamine the cases with AI assistance.

### Statistical analysis

After all panel members completed the grading of the biopsies, we compared their raw scores to the consensus reference standard. Scores were given on a six-point scale: 0 for benign, and 1–5 for grade groups. Cohen’s kappa with quadratic weights was used as the primary metric of performance. On a group-level, we used the median kappa as the metric to account for outliers.

To compare reading cases with or without AI assistance we conducted a statistical analysis, using the difference in kappa between the two reads as the test statistic. A Shapiro–Wilks test for normality was performed to show that the data were not normally distributed. To compare the difference in kappa scores, we performed a Wilcoxon signed-rank test on the paired kappa values. The test statistic was computed using the grades of the 100 cases that were used in both reads.

To account for possible bias in the reference standard, we computed the pairwise agreement between all panel members individually. The reference standard was not used in this analysis. Agreement was calculated using quadratically weighted Cohen’s kappa on the grade group.

In addition to a comparison of grade group agreement, we compared the concordance on estimated tumor volume. For each panel member, we computed Pearson’s correlation on the reported tumor volume with all other panel members (pairwise combinations). Correlations were computed on data of the 80 malignant cases that were used in both reads.

For the external Imagebase dataset, we used agreement using quadratically weighted Cohen’s kappa as the main metric, to allow for a comparison between the internal and external data. Agreement was calculated using linear weights in the Imagebase study [22], so we additionally computed the agreement using linear weights to compare between the two studies. As there was no consensus between Imagebase panel members for every case, we computed the average agreement of each panel member of the study pairwise with the Imagebase panel members, following similar work on this dataset [8]. To compare between reads, the median value of the pairwise kappas was used. A Shapiro–Wilks test for normality was performed to show that the data were not normally distributed. To compare the difference in kappa scores, we performed a Wilcoxon signed-rank test on the paired kappa values.

All statistical analyses were performed using Python (version 3.7.6), the pandas package (version 1.0.1), the scikit-learn package (version 0.22.2) and the SciPy package (version 1.4.1). Figures were generated using the matplotlib package (version 3.1.3).

## Results

### The observer panel

We invited 16 pathologists (board certified or residents) who participated in an earlier study on automated Gleason grading [7] to perform this observer experiment. Two panel members dropped out due to other obligations or a lack of time. In total, the observer panel consisted of 14 members (11 certified pathologists and 3 pathology residents), originating from 12 independent labs and 8 countries. All panel members had prior experience with Gleason grading, though with varying amounts of experience.

### Dataset under review and reference standard

From the test set that was previously used to evaluate our deep learning system [7], a set of 160 cases was selected to be reviewed by the panel. All cases under review had been graded by three uropathologists with extensive (>20 years) experience in Gleason grading, and their consensus opinion set the reference standard. The agreement between the uropathologists in the first round of the consensus-protocol was high (quadratic weighted Cohen’s kappa 0.925).

Of the selected cases, 100 cases were already graded by the panel as part of the previous study and were reused for the current study; the remaining 60 cases were unseen to act as controls, to measure the potential effect of a second-read on the original cases. The complete set of 160 cases for the AI-assisted read in the present study consisted of 30 (19%) benign cases, 22 (14%) cases with grade group 1, 26 (16%) cases with grade group 2, 32 (20%) cases with grade group 3, 20 (13%) cases with grade group 4 and 30 (19%) cases with grade group 5.

### AI-assisted gleason grading

After grading, all panel members filled in a questionnaire on the grading process. Five out of 14 (36%) panel members predicted that they scored somewhat better in comparison with the first read. Of these five, 3 out of 14 (21%) expected a performance increase due to being more experienced in viewing cases using the online viewer, and 2 out of 14 (14%) because of the AI assistance. The majority of the panel members (8 out of 14, 57%) indicated they did not expect a performance increase as a result of the AI assistance, while one pathologist (1 out of 14, 7%) expected to have scored somewhat lower.

**Fig. 2:**
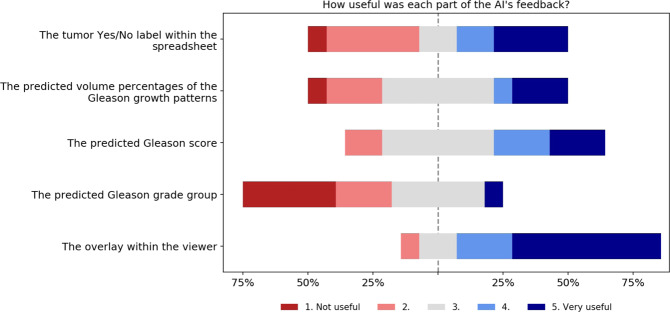
Survey results on the AI feedback. Panel members were asked to indicate how useful each part of the AI’s feedback was on a five-point scale from “Not useful” to “Very useful”.

**Fig. 3:**
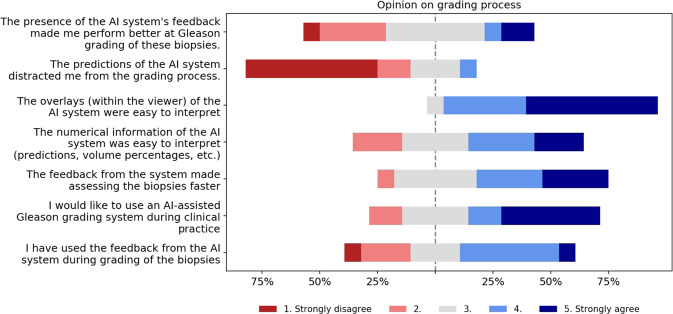
Survey results on the grading process. Panel members were asked to reflect on the grading process and answer questions on a five-point scale from “Strongly disagree” to “Strongly agree”.

Eleven out of 14 (79%) panel members indicated that they used feedback from the AI system during grading. Of all the components of the AI feedback, the Gleason pattern overlay was determined to be the most useful and easy to interpret (Fig. 2). The panel members indicated that the final grade group, as assigned by the system, was the least helpful. The majority of panel members noted that the AI assistance did not distract them from the grading process, but instead made grading the biopsies faster (Fig. 3).

### Performance of the panel with and without AI feedback

**Fig. 4:**
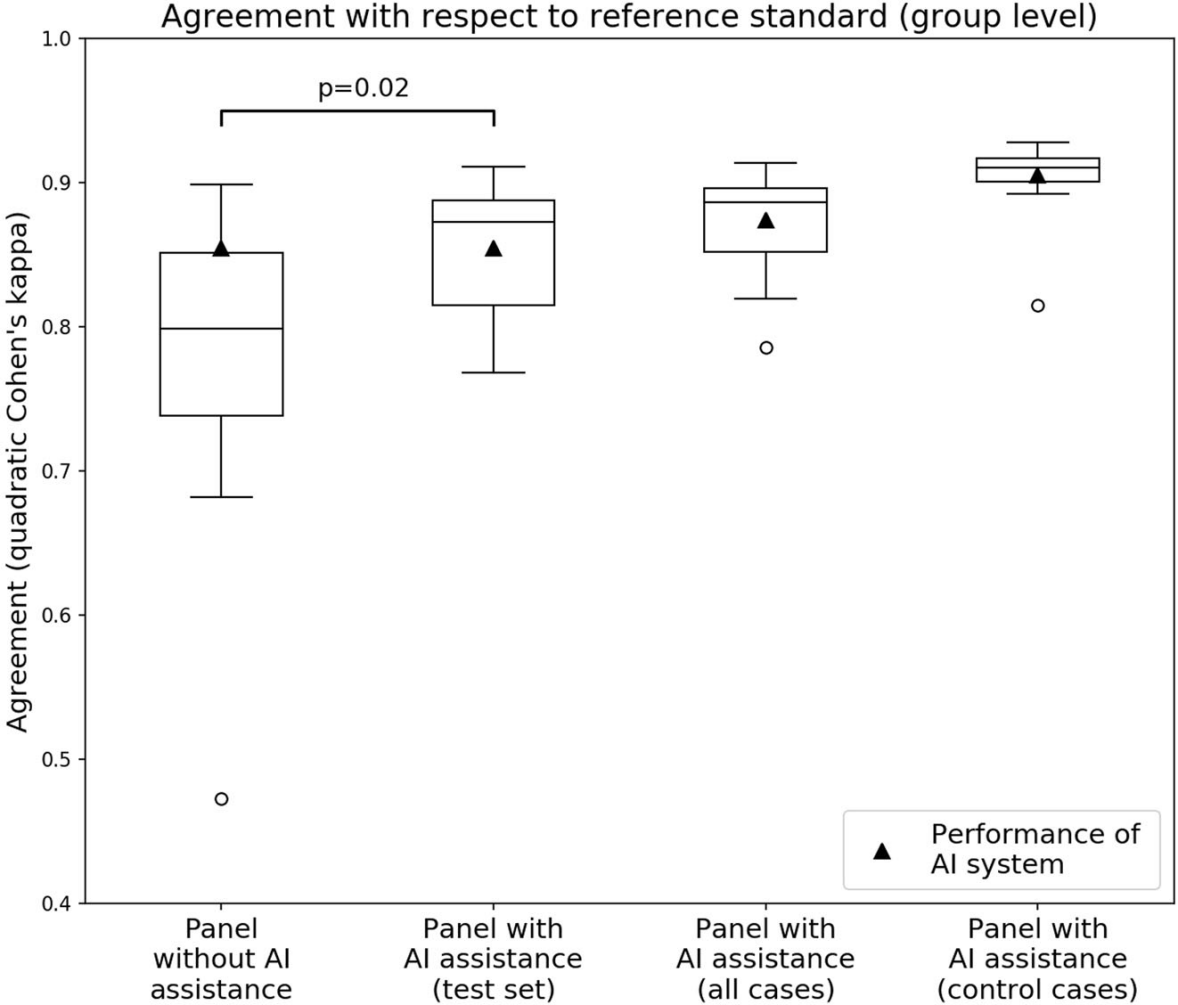
Panel performance with and without AI assistance. With AI assistance, the median performance of the group increased while the variability between panel members went down.

In the first read without AI assistance, the agreement with the reference standard (measured by the median quadratically weighted Cohen’s kappa) for the panel was 0.799. In the second, AI-assisted read, the median kappa of the panel increased to 0.872 (9.14% increase), showing a significant increase in performance (Wilcoxon signed-rank test *p* = 0.019, Fig. 4). On the same dataset, the AI system in itself achieved a kappa score of 0.854. Excluding panel members who estimated that they improved due to viewing more cases (*n* = 3) or excluding pathologists who indicated that they did not use the AI feedback (*n* = 3), we found a comparable increase in median kappa from, respectively 0.754–0.875 (*p* = 0.041) and 0.754–0.870 (*p* = 0.016).

Nine of the 14 (64%) panel members scored higher in the assisted read, while five (36%) panel members scored slightly lower, though with a maximum decrease in kappa score of 0.013. Of the five that scored lower, four already outperformed the AI system in the first read. The interquartile range of the panel’s kappa values dropped from 0.113 to 0.073 in the second read (Fig. 4).

**Fig. 5:**
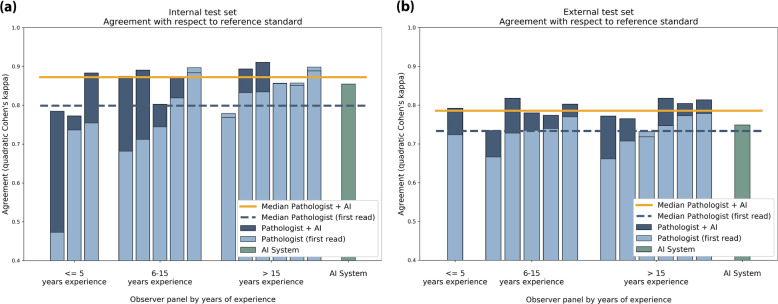
Individual performance of panel members shown for both the unassisted read (light blue) and assisted read (dark blue). Results for the internal test set shown in (**a**) and external test set shown in (**b**). Lower performance in the unassisted read is indicated with a line in the light blue bars. Pathologists are sorted based on experience level and the kappa value of the unassisted read. The performance of the standalone AI system is shown in green. In the unassisted reads, the AI system outperforms the group. In the assisted reads, the median performance of the group is higher than of the standalone AI system.

In the first read, the kappa value of the AI system exceeded that of 10 out of the 14 (71%) panel members. In the AI-assisted read, only five of the panel members (36%) scored a kappa value below that of the AI system. The largest improvement was seen for panel members who had less than 15 years of experience (Fig. 5a). Of the panel members who scored lower than the AI system in the unassisted read (10 out of 14, 71%), nine scored higher in the assisted read (9 out of 10, 90%). None of the panel members who outperformed the AI in the unassisted read improved in the assisted read. On a group-level, the median performance of AI-assisted reads was higher than both that of the standalone AI system and the unassisted reads.

The agreement of the panel with the reference standard on the control cases was high, with a median kappa value of 0.910, and slightly higher in comparison with the test cases (kappa 0.872). The control cases were only viewed in the assisted read. The system’s performance on the control cases was also higher, with a kappa value of 0.905 compared with 0.854 on the test cases.

**Fig. 6:**
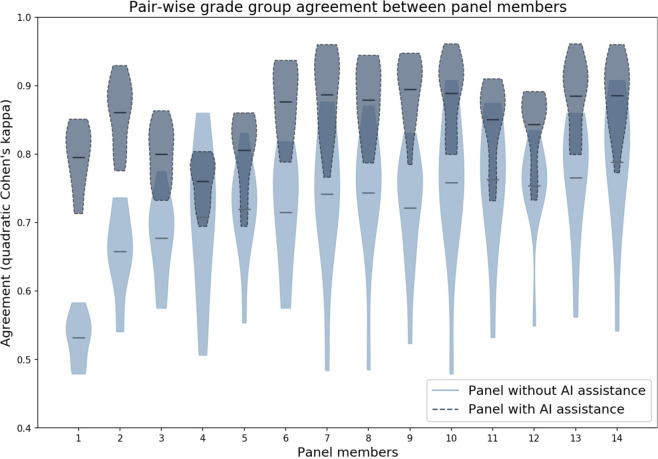
Pairwise agreement for each panel member with the other panel members. Each horizontal bar indicates the average agreement. The consensus reference standard was not used in this figure. The agreement in the assisted read (dark blue) is higher than in the unassisted read (light blue). Panel members are sorted based on their agreement in the unassisted read.

Between panel members, grading became more consistent in the assisted read. In the unassisted read, without taking the consensus into account, the median pair-wised agreement within the panel was 0.737 (quadratically weighted Cohen’s kappa). In the assisted read, this agreement increased to 0.859 (Fig. 6).

**Fig. 7:**
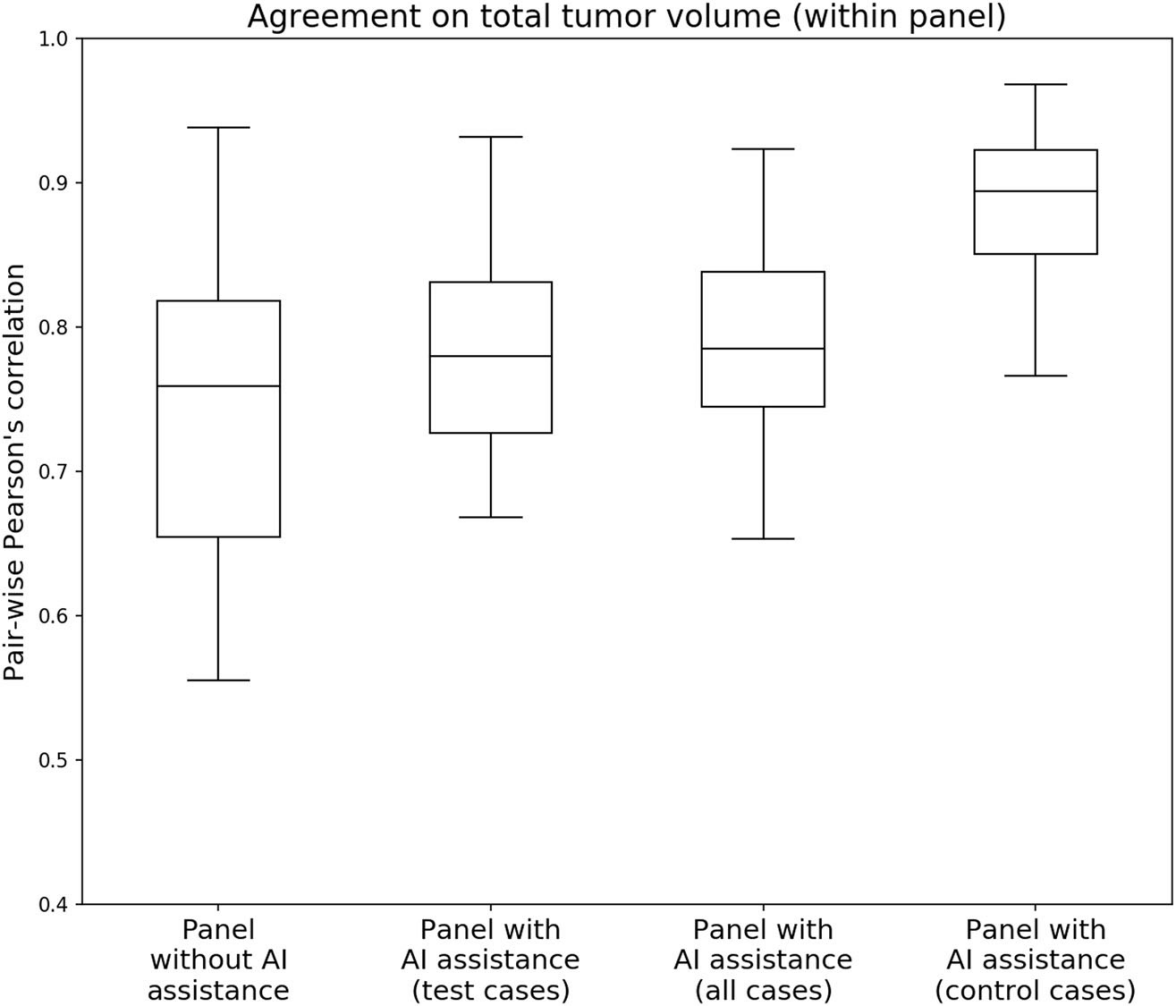
Pairwise correlations on reported total tumor volume between panel members. While only a slight increase can be observed in the assisted read, the total variation dropped substantially.

One pathologist did not report total tumor volume in the unassisted read and was excluded for the analysis of reported tumor volume. The correlation between the remaining panel members (13 out of 14) on total tumor volume was high in the unassisted read (mean Pearson’s *r* = 0.744), and increased slightly the assisted read (*r* = 0.780). Variation between panel members was lower in the assisted read; the interquartile range decreased from 0.164 to 0.105 (Fig. 7).

### External validation

For the Imagebase experiment, three cases and scores from one of the expert pathologists were excluded following the procedure by Ström et al. [8]. The remaining 87 cases all contained tumor, and the Imagebase panel had an average pairwise Cohen’s kappa of 0.819 (quadratically weighted) and 0.677 (linear weighted). The deep learning system achieved an average pairwise agreement of 0.748 (quadratically weighted) and 0.584 (linear weighted) with the Imagebase panel.

Of the 14 panel members, 12 joined the second experiment using the Imagebase dataset, two pathology residents dropped out due to other obligations. The remaining members graded all the cases in both reads. In the unassisted read, the median pairwise agreement with the Imagebase panel was 0.733 (quadratically weighted Cohen’s kappa) and 9 out of the 11 panel members (75%) scored lower than the AI system. Using linear weights, the agreement of the panel was 0.576.

With AI assistance, the agreement increased significantly to 0.786 (*p* = 0.003), with the majority of the panel now outperforming the standalone AI system (10 out of 12, 83%). Improvements could be seen for all but one of the panel members, with no clear effect of experience level (Fig. 5b). Measured using linear weighted Cohen’s kappa, the agreement in the assisted read increased to 0.631.

## Discussion

To the best of our knowledge, this study was the first to explore the possible merits of AI assistance on histological tumor grading. In a research setting, we showed that AI assistance improves pathologists’ performance at Gleason grading of prostate biopsies. Measured through the agreement with an expert reference standard, the read with AI assistance resulted in a significant increase in performance on the internal test set (quadratically weighted Cohen’s kappa, 0.799 vs. 0.872).

On an external validation set, *Imagebase*, the same positive effect of AI assistance was shown. With respect to a panel of international experts in prostate pathology, agreement increased from 0.733 to 0.786 (quadratically weighted Cohen’s kappa). In comparison with the internal set, the panel and AI system both scored lower on this external set. This could be explained due to several reasons: the Imagebase cases were collected specifically to represent a wide range of tissue patterns and the panel who set the reference standard reached consensus in only 50 of the cases, showing significant difficulty in the cases. Second, some differences can be accounted to the reference standard being set in 2015 and the use of microphotographs instead of whole-slide images by the expert panel. Last, the external dataset was collected in a different lab and scanned using a different scanner, which could negatively influence the accuracy of the feedback provided by the AI system.

Variance between panel members’ performance decreased due to AI assistance, resulting in overall more consistent grading. This decrease in grader variability was observed in comparison with the reference standard, and between panel members on both grade group and tumor volume estimation. Reduced observer variability of Gleason grading is highly desirable, as it could lead to a stronger prognostic marker for individual patients and reduces the effect of the diagnosing pathologist on potential treatment decisions.

In the unassisted read, the AI system outperformed 10 out of 14 pathologists, and this dropped to only 5 out of 14 in the second-read with AI assistance. Pathologists assisted by the AI system not only improved compared with unassisted reads but also achieved higher median performance than the standalone AI. These results indicate that there is a potential benefit of pathologists using AI assistance as a supportive tool during diagnosis. Especially in geographic regions where the number of pathologists is limited or subspecialized pathologists are not available, AI systems such as ours can support pathologists in achieving higher grading accuracy and consistency.

The most substantial increase in performance was seen for panel members who initially scored lower than the AI system. Most of the pathologists with more than 15 years of experience, who often outperformed the AI system in the unassisted read, scored comparably in both reads. Some pathologists’ scores approached the agreement between the pathologists who set the reference standard. In such cases, given the subjective nature of Gleason grading, objective improvement is difficult to determine. In the external set, which had a higher case difficulty, AI assistance improved the scores of all but one of the panel members.

While no performance gain was found for some pathologists in terms of diagnostic accuracy, most pathologists indicated that the use of the AI system led to faster grading. However, in this study, we did not directly measure the time taken per case nor did we limit the maximum time per case. For clinical applications, where reducing the workload and overall efficiency is an important topic, saving time through AI assistance is of great interest. Additional research could quantitively test the ability of AI systems to reduce time needed per case.

Through a questionnaire, we investigated the pathologists’ experiences when using the system. One of the design goals of the deep learning system was to support the workflow of pathologists. The system was developed to give feedback on multiple abstraction levels, with the grade group giving an overall assessment and the overlay more detailed feedback. We assumed that the precise gland-level segmentations of the tumor and Gleason patterns could support pathologists in quickly assessing glandular regions and assisting in volume measurements. Almost all pathologists indicated that the AI system’s overlay was useful, and, based on the questionnaire, was the most used feature of the system. AI assistance through these overlays can be seen as another tool for pathologists that gives feedback on a glandular level, comparable with, e.g., immunohistochemistry, and gives direct support for the systems’ case-level prediction. The overlays allow pathologists to combine their expertise with the added feedback of the system to determine the final grade.

The AI feedback was also given on a case-level through volume measurements, the Gleason score, and the grade group. Of all features, panel members rated the biopsy level grade group as the least useful. Given that the grade group is directly computed from the Gleason score and all feedback was presented at the same time, it can be seen as redundant information in the feedback.

While the results of this observer experiment are promising, several limitations have to be addressed. First, we cannot entirely exclude that factors outside of the AI feedback influenced the pathologists’ performance, both positively and negatively. While pathologists did not receive any feedback between the two reads, more experience with viewing cases digitally, the viewer, or in Gleason grading itself could have some influence on the results. Though, we believe that the influence of a second-read is small for several reasons: the majority of pathologists predicted that they scored the same, a significant increase was still found when excluding pathologists who indicated more experience, and the performance on the unseen control cases was also high. Furthermore, AI assistance also improved grading on external data, 6 months after the first experiment, which would be unlikely if the measured effect could be contributed to a higher experience level. For future research, the order in which pathologists received AI assistance could be randomized to further exclude these factors.

Secondly, pathologists were not extensively trained or instructed to use the AI system and were free to use the system in any way during grading. All cases that were graded were also included in the analysis, and we did not allow for a training phase. Pathologists can benefit from an understanding of the global properties of an AI system when such a system is introduced in their grading process; this includes the system’s limitations, its tendency to over- or under grade, and the overall design goal [23]. A training phase at the start of the observer experiment could have increased the use, understanding, and effectiveness of the AI feedback during the grading process and might have led to further increased performance by using the AI system.

Third, in this study, we focused on the assessment of individual biopsies whereas in clinical practice pathologists will examine multiple biopsies per patient. The dataset used to develop the AI system only included one glass slide per patient and the pathologists who set the reference standard evaluated each biopsy individually. An important avenue for future research would be to investigate AI assistance on a patient-level, which allows for new approaches such as automatically prioritizing slides.

Last, the selection of cases under review can influence overall results. We performed our main experiment using a limited set of 100 test and 60 control cases from a single center in a research setting. The results on the external validation set showed that AI assistance still improved grading, even on data with a different case distribution and reference standard. Nonetheless, for clinical validation of AI tools and their benefit to day-to-day practice, more cases, collected under different settings and from additional centers, should be included.

To the best of our knowledge, our study is the first to show the benefit of AI support for Gleason grading. Ultimately, additional research should determine whether the added benefit of AI assistance results in a stronger prognostic marker for individual patients.
